# Comparison of genomic predictions for carcass and reproduction traits in Berkshire, Duroc and Yorkshire populations in Korea

**DOI:** 10.5713/ajas.18.0672

**Published:** 2019-07-01

**Authors:** Asif Iqbal, Tae-Jeong Choi, You-Sam Kim, Yun-Mi Lee, M. Zahangir Alam, Jong-Hyun Jung, Ho-Sung Choe, Jong-Joo Kim

**Affiliations:** 1 Department of Biotechnology, Yeungnam University, Gyeongsan 38541, Korea; 2 Swine Science Division, National Institute of Animal Science, RDA, Wanju 55365, Korea; 3 Jung P&C Institute, Seoul 16950, Korea; 4 Department of Animal Biotechnology, Chonbuk National University, Jeonju 54896, Korea

**Keywords:** Genomic Selection, Genomic Estimated Breeding Value (GEBV), Genomic Best Linear Unbiased Prediction (GBLUP), Carcass Traits, Reproduction Traits, Pig

## Abstract

**Objective:**

A genome-based best linear unbiased prediction (GBLUP) method was applied to evaluate accuracies of genomic estimated breeding value (GEBV) of carcass and reproductive traits in Berkshire, Duroc and Yorkshire populations in Korean swine breeding farms.

**Methods:**

The data comprised a total of 1,870, 696, and 1,723 genotyped pigs belonging to Berkshire, Duroc and Yorkshire breeds, respectively. Reference populations for carcass traits consisted of 888 Berkshire, 466 Duroc, and 1,208 Yorkshire pigs, and those for reproductive traits comprised 210, 154, and 890 dams for the respective breeds. The carcass traits analyzed were backfat thickness (BFT) and carcass weight (CWT), and the reproductive traits were total number born (TNB) and number born alive (NBA). For each trait, GEBV accuracies were evaluated with a GEBV BLUP model and realized GEBVs.

**Results:**

The accuracies under the GBLUP model for BFT and CWT ranged from 0.33–0.72 and 0.33–0.63, respectively. For NBA and TNB, the model accuracies ranged 0.32 to 0.54 and 0.39 to 0.56, respectively. The realized accuracy estimates for BFT and CWT ranged 0.30 to 0.46 and 0.09 to 0.27, respectively, and 0.50 to 0.70 and 0.70 to 0.87 for NBA and TNB, respectively. For the carcass traits, the GEBV accuracies under the GBLUP model were higher than the realized GEBV accuracies across the breed populations, while for reproductive traits the realized accuracies were higher than the model based GEBV accuracies.

**Conclusion:**

The genomic prediction accuracy increased with reference population size and heritability of the trait. The GEBV accuracies were also influenced by GEBV estimation method, such that careful selection of animals based on the estimated GEBVs is needed. GEBV accuracy will increase with a larger sized reference population, which would be more beneficial for traits with low heritability such as reproductive traits.

## INTRODUCTION

The application of molecular genetics to livestock breeding began in the early 1970’s, followed by the introduction of microsatellite markers in early 1990’s, which allowed the use of DNA marker information to identify quantitative trait loci (QTL) associated with production traits, as well as to make selection decisions at an early age through marker-assisted selection (MAS) [1]. So far, substantial numbers of QTLs and candidate genes have been reported from many QTL mapping studies (www.animalgenome.org) [2]. Nevertheless, the implementation of MAS in livestock has been limited, because most of the economically important traits are polygenic in nature, where many loci with small effects contribute to the phenotype. Thus, the use of few markers throughout the genome could explain only small proportion of the total genetic variance, leaving much of the genetic variance of the trait unexplained [3].

Recently, rapid development of DNA genotyping technology has enabled genotyping of animals with high-density markers. With new sophisticated statistical models, genomic regions or QTL have been more efficiently identified that could be incorporated into MAS in livestock breeding, in order to enhance selection response per generation [4]. This suggests for the application of genome-wide high-density markers to capture most of the genetic variance of the traits of interest.

Meanwhile, high-throughput single nucleotide polymorphism (SNP) genotyping technologies with genome-wide high-density SNP arrays are available, such that at least one SNP marker could be in linkage disequilibrium with QTL underlying a trait of interest, enabling predicting of genomic estimated breeding values (GEBVs). GEBV prediction involves estimation and summation of the effects of all markers throughout the genome [5]. Genomic selection (GS) that is based on GEBVs allows selecting animals at an early age, which reduces generation interval and increase rate of gain over traditional approaches [6]. The GS is very promising for genetic improvement of the traits that are sex-limited, difficult or expensive to measure, or lowly heritable [3].

There are several approaches to GEBV prediction, such as genome-based best linear unbiased prediction (GBLUP) or Bayesian methods. The proposed statistical models have different assumptions about distribution of QTL effects, number of QTL fitted in the model, etc. In GBLUP, the statistical model assumes that a very large number of QTLs affect a trait, where each of the QTL has a small effect with equal variances under a normal distribution. On the contrary, the Bayesian statistical model assumes that QTLs effects are distributed with unequal variances which follow *t* distributions [5]. However, violation of the assumptions under the given models would cause biased prediction of GEBVs.

In GS, evaluation of animals is based on their GEBVs. Unlike cattle, the benefit of GS in pigs would not be substantially improved by shortening the generation interval. Instead, accurate prediction of GEBVs would play pivotal roles in enhancing selection response in pigs [7]. The accuracy of GEBVs is affected by several factors such as methods of GEBV prediction, size of the training population, effective population size (Ne), effective number of chromosome segments which is a function of Ne, heritability, the proportion of genetic variance at causal variants captured by observed SNPs, genetic correlation between reference and target samples and marker density [8,9]. Although, there are several reports of successful implementation of GS for genetic improvement of traits in dairy cows [10,11], there are few reports of GS studies in pigs [5,9]. Herein, we report the evaluations of GEBV accuracy for carcass and reproductive traits in Berkshire, Duroc, and Yorkshire populations, compare accuracies between traits or breed populations, and to compare GEBV accuracies between a BLUP model and realized GEBVs.

## MATERIALS AND METHODS

### Animal management and phenotypes

For carcass traits, we collected a total of 1,870, 696, and 1,723 pigs belonging to Berkshire, Duroc, and Yorkshire, respectively, with the pigs being born between the year 2007 and 2015. Similarly, data on 1,487, 643, and 914 pigs born between 2011 and 2015 were recorded for the two reproductive traits (number born alive [NBA] and total number born [TNB] for first three parities), pertaining to the respective pig breeds. The averages of the three parities were used for GEBV prediction. All Berkshire pigs were collected from Dasan breeding farm, in Namwon, Jeonnam, whereas the Duroc and Yorkshire samples were collected from Nong-Hyup breeding farms in Youngwang, Jeonnam, Korea.

The pigs were born from 52 sires and 570 dams for Berkshire, from 141 sires and 319 dams for Duroc, and from 87 sires and 643 dams for Yorkshire, respectively. The piglets were weaned at three to four weeks of age and moved into piglet pens, in each of which about 100 piglets were raised for 60 days. The pigs were then placed in growth/fattening pens with the size of 20 pigs for 90 to 120 days. The pigs were fed with commercial feeds according to the regimen of Purina Ltd in Nonghyup commercial farms, Youngsam, Chounbuk, Korea. The individuals were then transported to nearby abattoirs and slaughtered. All records of the pedigrees, genotypes and phenotypes were provided by the Nonghyup breeding company not explicitly for the purpose of this study.

The average slaughter ages were 208±18, 131±32, and 155± 10 days for Berkshire, Duroc and Yorkshire breeds, respectively. Hot carcass weight was measured immediately after slaughter on the floor of the abattoir. The carcasses were then cooled in a chilling room maintained at 0°C for 24 hours. The average of backfat at the first rib, last rib, and lumber vertebra was calculated with help of slide calipers [12].

For the reproductive traits, the numbers of piglets that are born alive (NBA) and total number of piglets born (TNB) were recorded for the first three parities, and the average of the three parities was calculated for the two traits.

### Genotyping and quality control

The pigs were genotyped with Illumina Porcine SNP60K BeadChip (Illumina Inc, San Diego, CA, USA), on which 62,163 SNPs were embedded. All autosomal SNPs were used for quality control (QC) procedures. The SNPs were removed with call rates <90%, minor allele frequency <1%, and significant deviation from Hardy-Weinberg equilibrium at p< 0.000001. The individuals with genotyping call rate <90% were also deleted. PLINK software (v1.07) was used for the QC procedures [13]. After the QC tests, the genome-wide missing SNPs were imputed with the use of Beagle vs 3.3.2 [14]. Identity-by-state test was also carried out to check whether there are similar individuals or genotyping error in the datasets. The pairs of individuals with high similarity rate (>95%) were removed for GEBV evaluation.

### Statistical analysis

The statistical significance of the fixed factors or covariates was tested using SAS general linear model procedure (SAS, version 9.2, SAS Institute, Cary, NC, USA) for fitting the factors into Animal model. For carcass traits, gender and slaughter age were fitted as a fixed effect and a covariate, respectively. For the reproduction traits, only birth year-season was fitted as a fixed effect in the animal model for ASREML analysis [15]. The linear mixed model for each trait is:

y=Xb+Zg+e

Where *y* represents vector of the phenotypic records for *n* number of animals, **X** denotes the design matrix of the fixed effects and/or covariates, *b* is the vector of the corresponding effects including overall mean, **Z** is the design matrix assigned to genomic breeding values, *g* is the vector of the breeding values, and *e* is the vector of residual error, which is assumed to be normally distributed with $\sigma_{e}^{2}$.

In matrix notation, the mixed model equation is written as:

$$\left[\begin{array}{cc} X^{\prime }X & X^{\prime }Z\\ Z^{\prime }X & Z^{\prime }Z+G^{-1}\alpha \end{array}\right]\;\left[\begin{array}{c} \hat{b}\\ \hat{g} \end{array}\right]=\left[\begin{array}{c} X^{\prime }Y\\ Z^{\prime }Y \end{array}\right]$$

Where, $\alpha =\frac{\sigma_{e}^{2}}{\sigma_{a}^{2}},\;\sigma_{a}^{2}$ is the genetic variance, and $\sigma_{e}^{2}$ is the error variance.

By solving the MME, the *ĝ*, breeding values could be obtained as:

$$\left[\hat{g}]={\left[Z^{\prime }Z+G^{-1}\alpha \right]}^{-1}[Z^{\prime }Y\right]$$

The genomic relationship matrix (**G**) matrix was built using the genome-wide complex trait analysis (GCTA) tools [16]. The following equation was used to generate **G** matrix based on marker allele frequencies:

$$G=\frac{(M-P){\left(M-P\right)}^{\prime }}{2\;\Sigma_{j=1}^{m}\;P_{j}(1-P_{j})}$$

Where, **M** has dimensions of n×m, *n* is the number of individuals and *m* is the number of markers used. The genotypes of each marker are coded as AA = −1, AB = 0, BB = 1 for alternate alleles, A and B. In the **P** matrix, an element, *P*_j_ = 2(*P*_j_–0.5), where *P*_j_ represents minor allele frequency at locus j. (***M***–***P***) represents incidence matrix (**Z**) for the markers.

### Prediction of GEBV and evaluation of GEBV accuracy

After estimating marker effects, GEBV of an animal *i* was calculated using the following equation:

$${\text{GEBV}}_{i}=\Sigma_{j=1}^{m}\;z_{ij}\;{\hat{g}}_{j}$$

Where, *z_ij_* denotes the genotype of *i^th^* individual at the *j^th^* marker locus, and *ĝ_j_* represents allele substitution effect of the *j^th^* marker.

GEBV accuracies were evaluated by applying the standard error of GEBVs [6] obtained from ASREML output. The following calculation formula was used to estimate GEBV accuracies for an animal *i*.

$$\text{Model }{\text{accuracy}}_{i}=\sqrt{(1-stderror{\left(g_{i}\right)}^{2}/\sigma_{a}^{2}}$$

Where, $\sigma_{a}^{2}$ represents the genetic variance of the trait. Realized accuracies were also obtained with a replicated training-testing method via a 10-fold cross-validations method according to Badke et al [17]. The entire dataset was divided into 10 groups, in which one of the groups (10% animals) was treated as testing, i.e. the samples were assumed to have only genotype information and their phenotypes masked, while the remaining samples (90% of animals) was used as training or reference group with both genotype and phenotype known, to predict GEBVs in the test group. This cycle was repeated 10 times such that each of the animals in the dataset could get the chance to be treated once as a testing group. GEBV accuracies of the testing samples were measured by the correlations between the GEBV estimates and real phenotype values as follows:

Realized accuracy=r (GEBVs,y)

Where, *r* is Pearson’s correlation coefficient, and *y* is individual phenotype.

Genetic and residual variances were also estimated with the use of ASREML 3.0 program [17]. The heritability values for BFT, CWT, NBA, and TNB were calculated for each breed using the following formula:

$$h^{2}=\frac{\sigma_{a}^{2}}{\sigma_{a}^{2}+\sigma_{e}^{2}}$$

## RESULTS AND DISCUSSION

### Phenotypes and genotypes

The pigs were slaughtered at 131 to 209 days with the highest slaughter age for Berkshire pigs, which might be due to its slow rate of growth (Table 1) [18]. The mean carcass weight ranged 84 to 103 kg with the lowest weight for Berkshire. Also, the Berkshire pigs yielded the greatest (23.8 mm) BFT, while the Duroc pigs had the lowest BFT (13.6 mm), which was in accordance with the previous reports [19]. For carcass weight, there was the greatest variation between individuals in the Duroc samples, while the Berkshire samples had the most various BFT measurements among the three breeds (Table 1).

After QC tests, the numbers of available SNPs for the carcass traits were 30,769, 26,374, and 30,071 in Berkshire, Duroc, and Yorkshire samples, respectively. Also, 39,095, 47,251, and 52,258 SNPs were available for the reproductive traits in the respective breeds. Among the SNPs, the 25,880 common SNPs were finally chosen for prediction of GEBVs, for which the average distance between adjacent SNPs across the autosomal chromosomes was 94.3±143.4 kb, covering 2,437.9 Mb of the porcine genome (results not shown).

### Estimation of genetic parameters

Additive genetic and residual variances, and heritabilities were estimated under the GBLUP model for the carcass (Table 2) and the reproductive traits (Table 3), respectively. The heritabilities for BFT and CWT ranged 0.24 to 0.50 and 0.25 to 0.43, respectively, for the three pig breeds. In the Berkshire and Duroc populations, the BFT heritability estimates were 0.50 and 0.41, respectively. This result agreed with the previous reports [20,21], in which the estimated heritabilities of the trait ranged from 0.34 to 0.56 for the same breeds. For CWT, the estimated heritabilities of 0.25 to 0.43 for the three breeds fell within the range (0.13 to 0.43) of heritability estimates in previous reports [7,20]. These results show that the use of genome relationship matrices in heritability estimation of carcass traits in pigs were, in general, in good agreement with the use of pedigree-based relationship matrices.

The heritability estimates for NBA and TNB in the three breed populations ranged 0.03 to 0.32 and 0.15 to 0.46, respectively (Table 3). The lowest heritability values were observed in the Berkshire population (0.15 and 0.03 for TNB and NBA), while the greatest in the Duroc population (0.46 and 0.32 for TNB and NBA). In general, NBA has been reported to have very low heritability, i.e. <0.10 [22], which was in good agreement with the estimate in Berkshire in this study (Table 3). However, in Duroc, the heritability estimates for the reproductive traits were high, which may be partly due to a small sample size (Table 1). The TNB heritability was estimated as 0.15 in Berkshire, which was in good agreement with the heritability value in commercial pig populations reported by Cleveland et al [23] and Cleveland and Hickey [24]. In the three breed populations, heritability values for TNB were greater than those for NBA, which was also agreed with the previous reports [22,24].

### Evaluation of GEBV prediction accuracies for carcass traits

The BLUP model accuracies of GEBV estimates of BFT were 0.59 and 0.72 for Yorkshire and Berkshire, respectively, and 0.59 and 0.63 for CWT in the respective pig breeds. However, in Duroc pigs, the model accuracies of GEBV were 0.33 for both BFT and CWT, which was lower than the former two breeds (Table 4). This may be partly due to a smaller training sample size of Duroc (n = 466), compared to those of Berkshire (888) and Yorkshire (1,208).

The GEBV accuracies for the BFT and CWT in Berkshire agreed with Baby et al [20], who reported 0.68 and 0.60 for the respective traits under a GBLUP model in Berkshire. Also, several studies reported that BFT GEBV accuracies in the three pig breeds ranged 0.58 to 0.86 under various GEBV models [17,25,26].

The realized accuracies of GEBV estimates were in general lower than the model based accuracies for the carcass traits. In the Duroc population the realized accuracy for CWT was 0.09, while the model accuracy of the trait was 0.33, which might be partly due to a sampling effect of the small reference size of the breed (Table 4).

### Evaluation of GEBV prediction accuracies for reproduction traits

The results of the GEBV prediction accuracies for NBA and TNB in Berkshire, Duroc, and Yorkshire pigs are presented in Table 5. The accuracies obtained from the BLUP model ranged 0.32 to 0.54 and 0.39 to 0.56 for NBA and TNB, respectively. However, the realized accuracy estimates were higher than the model based accuracies, ranging 0.50 to 0.70 and 0.70 to 0.87, for the respective traits, which may be partly due to sampling effect, i.e. small sample size of the reference (training) population size.

The model accuracy estimate of TNB (0.56) in the Yorkshire population was in good agreement with Uimari et al [27] in Finish Yorkshire. Forni et al [28] applied single-step BLUP analyses to predict GEBV accuracies for TNB, resulting in the accuracy values of 0.28 to 0.49, overlapping the estimates of this study (Table 5). In general, the GEBV accuracies for TNB were higher than for NBA in the three breeds, which may be partly due to greater heritabilities of TNB than of NBA, because a high heritability tends to increase accuracy of genomic prediction [9].

The realized accuracies (0.70 to 0.87) of TNB agreed with Cleveland et al [24], in which the GEBV accuracies of the trait ranged 0.82 to 0.83 in a Landrace population with the sample size of 3,000 pigs. Both the model based accuracy estimates and the realized accuracy estimates were the greatest in the Duroc population (Table 5). This might be caused by the overestimation of the heritabilities, resulting from the very small reference population size in Duroc (Table 3).

In this study, GEBV accuracies for carcass and reproduction traits in pigs were evaluated based on two approaches, BLUP model-based or realized accuracy, i.e. correlation between predicted GEBVs and phenotypes. For the carcass traits, the model based accuracies were higher than the realized accuracies, while, for the reproductive traits, vice versa, across the three pig breed populations (Tables 4, 5). There are several factors influencing GEBV accuracies such as reference population size, heritability of trait, model assumptions, magnitude of genetic relatedness between training set and testing individuals, phenotype heterogeneity, density (or number) of the genome-wide markers [26]. Effective population sizes (Ne) were estimated for each breed according to Lee et al [9] that was based on effective number of chromosome segments and off-diagonal elements in genome relationship matrix, resulting in 162, 638, and 200 for Berkshire, Duroc and Yorkshire, respectively. Berkshire has a smaller Ne than Yorkshire, which may partly cause greater GEBV accuracies in carcass traits of the testing samples in Berkshire than in Yorkshire, even if the training sample size of Yorkshire was greater than Berkshire (Table 4).

It seems that the GEBV estimates of the carcass traits were less biased than those of the reproductive traits, because prediction of the estimates were based on greater reference population sizes and higher heritabilities (Tables 4, 5). For the two carcass traits (Table 4), the higher GEBV accuracies obtained in the model-based methods may be caused by the presence of individuals in the training set that are more genetically related to the selection candidates [25]. However, this result may be unexpected when the reference population size was too small, e.g. for the reproductive traits (Table 5), so that a small number (one tenth) of testing samples were so closely related to the rest (nine tenth) of the reference (training) samples, resulting in much higher realized accuracy.

Our results suggest that GEBV estimates for carcass and reproductive traits can be obtained with reasonable degree of prediction accuracy, even if small or moderate sizes of reference populations were used in the Korean breeding pig farms. However, much careful selection of animals based on the estimated GEBVs is needed, because GEBV accuracies could depend on alternate prediction methods. Instead, increase of GEBV accuracy can be warranted by incorporating large reference population sizes, e.g. several thousands of animals with both SNP genotypes and phenotypes, which would be more beneficial for low inheritable traits such as reproductive traits.

## Figures and Tables

**Table 1:** Summary statistics of two carcass trait measurements in Berkshire, Duroc and Yorkshire populations

Items	Carcass weight (kg)			Backfat thickness (mm)			Slaughter age (d)		
		
	Berkshire	Duroc	Yorkshire	Berkshire	Duroc	Yorkshire	Berkshire	Duroc	Yorkshire
Number of records	920	494	1,228	920	494	1,228	920	494	1,228
Mean (±SD)	83.9±5.9	102±10.8	103±8.6	23.8±5.0	13.6±2.4	14.8±0.25	209±17.3	131±32.7	155±10.6
Minimum	65	78	74	13.0	7.94	9.1	155	57	123
Maximum	104	149	136	43.0	24.9	23.1	286	186	206
CV (%)	7.0	10.6	8.4	21.0	17.3	16.9	8.3	24.9	6.8

SD, standard deviation; CV, coefficient of variation.

**Table 2:** Estimation of variance components and heritabilities for two carcass traits in Berkshire, Duroc, and Yorkshire populations

Items	Backfat thickness			Carcass weight		
	
	Berkshire	Duroc	Yorkshire	Berkshire	Duroc	Yorkshire
$\sigma_{a}^{2}\pm \text{SE}$	12.3±2.0	1.49±0.39	0.013±0.0	8.06±1.9	31.1±8.6	10.5±2.1
$\sigma_{e}^{2}\pm \text{SE}$	12.5±1.0	2.18±0.30	0.04±0.0	21.4±1.4	42.0±5.9	31.0±1.8
h^2^	0.50	0.41	0.24	0.27	0.43	0.25

SE, standard error.

**Table 3:** Estimation of variance components and heritabilities for two reproductive traits in Berkshire, Duroc, and Yorkshire populations

Items	Total number of born			Number born alive		
	
	Berkshire	Duroc	Yorkshire	Berkshire	Duroc	Yorkshire
$\sigma_{a}^{2}\pm \text{SE}$	0.78±0.6	1.76±1.0	0.89±0.2	0.014±0.4	1.48±1.3	0.71±0.2
$\sigma_{e}^{2}\pm \text{SE}$	4.36±0.6	1.96±0.8	2.41±0.18	4.75±0.6	3.11±1.1	2.3±0.2
h^2^	0.15	0.46	0.27	0.03	0.32	0.24

SE, standard error.

**Table 4:** Accuracies of GEBV prediction under the GBLUP model for carcass traits in the three pig breed populations

Items	Backfat thickness			Carcass weight		
	
	Berkshire	Duroc	Yorkshire	Berkshire	Duroc	Yorkshire
Model accuracy						
Training sample size	888	466	1,208	888	466	1,208
Testing sample size	982	230	515	982	230	515
GEBV accuracy	0.72	0.33	0.59	0.63	0.33	0.59
Realized accuracy						
Training sample size	800	420	1,088	800	420	1,088
Testing sample size	88	46	120	88	46	120
GEBV accuracy	0.46	0.37	0.30	0.27	0.09	0.17

GEBV, genomic estimated breeding value; GBLUP, genome-based best linear unbiased prediction.

Model accuracy was obtained from the GEBV BLUP model, which was functions of prediction error variance and genetic variances, while realized accuracy was calculated using correlation between GEBV estimates and phenotypic values.

**Table 5:** Accuracies of GEBV prediction under the GBLUP model for reproductive traits in the three pig breed populations

Items	Number born alive			Total number born		
	
	Berkshire	Duroc	Yorkshire	Berkshire	Duroc	Yorkshire
Model accuracy						
Training sample size	210	154	890	210	154	890
Testing sample size	1,277	489	24	1,277	489	24
GEBV accuracy	0.32	0.35	0.54	0.42	0.39	0.56
Realized accuracy						
Training sample size	212	154	890	212	154	890
Testing sample size	212	154	890	212	154	890
GEBV accuracy	0.50	0.64	0.70	0.70	0.87	0.72

GEBV, genomic estimated breeding value; GBLUP, genome-based best linear unbiased prediction.

Model accuracy was obtained from the GEBV BLUP model, which was functions of prediction error variance and genetic variances, while realized accuracy was calculated using correlation between GEBV estimates and phenotypic values.
